# Use of a hypogastric flap and split-thickness skin grafting for a degloving injury of the penis and scrotum: A different approach

**DOI:** 10.4103/0970-0358.59296

**Published:** 2009

**Authors:** S. Sengathir Selvan, Ganesh S. Alagu, R. Gunasekaran

**Affiliations:** Department of Plastic Surgery, Meenakshi Medical College and Research Institute, Kanchipuram, Tamil Nadu, India

**Keywords:** Hypogastric flap, penile degloving, scrotal degloving

## Abstract

Penile and scrotal skin avulsions are not common events and are caused usually by accidents with industrial machines or agricultural machines. We report a case of a 27-year-old newly married thin-built patient with avulsion and traumatic degloving of the penile and scrotal skin, with exposure of the corpora cavernosa and copus spongiosum of penis and testes as his loose clothes got entangled in a paddy harvesting machine accidently. Reconstruction was performed using a hypogastric flap and split skin graft, achieving a satisfactory aesthetic result and sexual functions.

## INTRODUCTION

Skin avulsions of male genitals are a rare plastic surgical emergency.[1] Although not life threatening, such lesions are incapacitating and psychologically devastating[2] and occur mainly because of accidents with industrial machines or agricultural machine belts.[2,3] Injuries at this site vary from simple lacerations to virtual emasculations.[1] Generally, wounds reach only the skin, causing minimal bleeding without producing damage to cavernous bodies, the spongy body or testes,[1,2] but the reconstruction part poses a real challenge. While a variety of methods have been described, reconstruction must not only achieve an aesthetic result but also maintain sexual function.

## CASE REPORT

**Figure 1:**
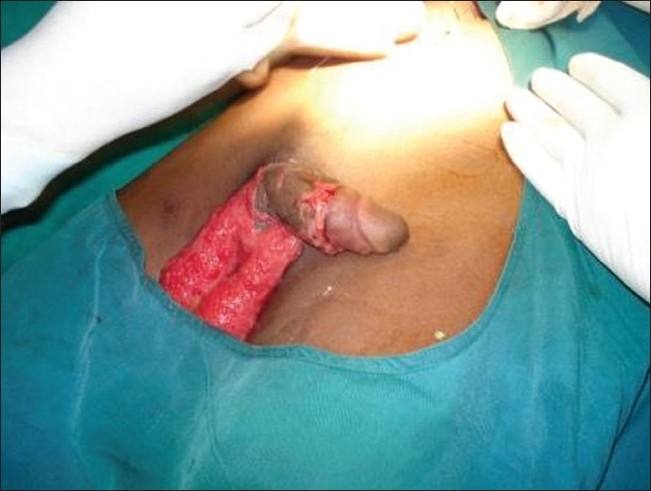
Degloving injury of penis and scrotum

A 27-year-old male was admitted to the general surgical department of our hospital following a degloving injury to his penis and scrotum [Figure 1]. He was working in a paddy field, when his clothes (dhoti) got entangled in the paddy harvesting machine resulting in complete avulsion and degloving of the penile and scrotal skin, with the exposure of the cavernous bodies, spongy body and testes.

The patient was resuscitated, and an emergency debridement was performed. and the penis was covered with a split skin graft. The bare testes were however left open with moist dressings and he was referred to our services for scrotal reconstruction.

**Figure 2:**
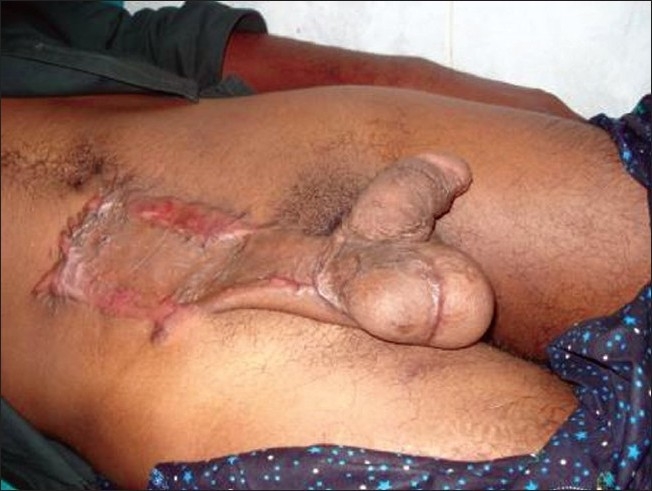
Hypogastric flap used to cover the posterior aspect of scrotum

A two stage abdominal flap with split skin graft was planned for scrotal reconstruction. A hypogastric flap measuring 15 × 10 cm, based on the superficial epigastric artery of was raised. The flap was attached to the posterior aspect of the scrotum and penoscrotal junction [Figure 2]. The remaining raw area of the scrotum, testes and the donor site was grafted with spilt skin graft harvested from the right thigh.

**Figure 3:**
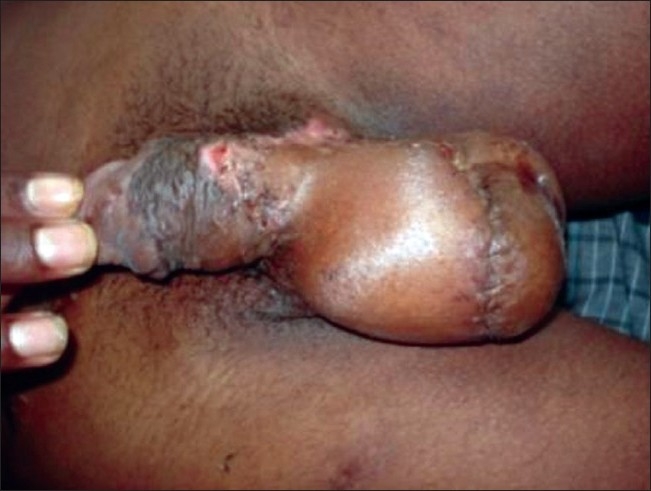
Flap fivision and inset in peno-scrotal junction

**Figure 4:**
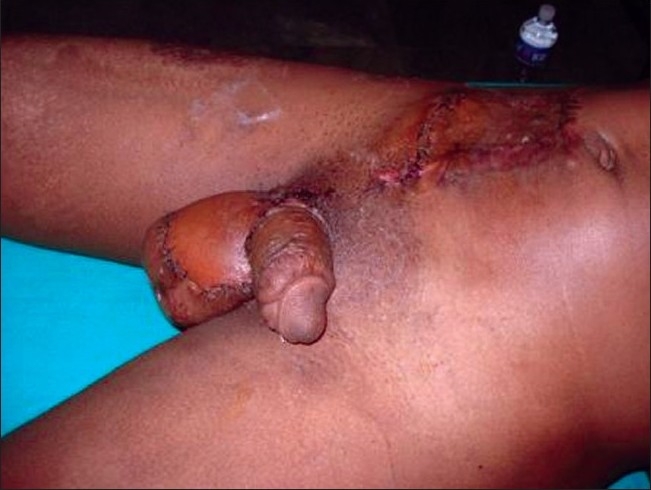
Remaining flap wrapped around the front of scrotum after pedicle division. Penile shaft is skin grafted

While the post-operative period was uneventful and the patient was advised flap division and inset after three weeks, he came back for follow-up only after 2 months. He had developed a contracture at the penoscrotal junction by then. We proceeded with releasing this contracture and the split skin grafting the anterior aspect of the scrotum. Division of the flap and inset was done [Figure 3, 4] covering the released penoscrotal junction and the anterior aspect of the scrotum. The final result was cosmetically very appealing and of great psychological benefit to the patient.

## DISCUSSION

Degloving injuries of the penis and scrotum are rare, if ever, life threatening. Less than 50% scrotal skin loss can often be closed primarily without difficulties immediately after trauma, with the surrounding tissue. In cases of a more significant skin loss, the testes may be preserved by placing them in thigh pouches or split-thickness skin grafting can be performed.[8]

Some surgeons prefer staged reconstruction with thigh pedicle flaps. In the case of medial thigh flaps, it involves bilateral subcutaneous elevation of the medial thigh skin and advancing towards the midline where both flaps are sutured to each other. Although this provides an effective coverage, the oval contour of the scrotal area is lost and may produce an unusual appearance to the region which may need to be revised.[4] Alternatively, one proximally based thigh flap in a single stage cannot cover both the penoscrotal and perineoscrotal junction. It needs two flaps. Moreover, the perineal cleft between the thigh and the scrotum will be obliterated and the oval contour of the scrotum will be lost.

Scrotal reconstruction by rapid intraoperative tissue expansion using tissue expanders has been done for partial loss of scrotal skin.[5] Experimental scrotal reconstruction has been done in rabbits using hypogastric flaps.[6] Whatever be the reconstruction, the psychological burden inflicted on patients by this type of injury is so great that they require increased attention and care by the plastic surgeon. There is a greater need for the restoration of form and function with this type of injury.[3] Spermatogenesis is not altered in the early stage (up to 3 months), but can be substantially abnormal in the late stage.[7] The thinner the skin cover, the better the spermatogenesis.[7]

Our patient had complete loss of both penile and scrotal skin with no remnants. He was thin built and was married 2 months back. He was psychologically deranged due to this trauma. The newly married couple could not accept the implantation of testis into the thighs as it is not cosmetically acceptable. Grafting of the penis and scrotum would produce contracture at the penoscrotal and perineoscrotal junction restricting the erection of the penis and mobility of the scrotum. We counseled both the patient and his wife and proceeded for the hypogastric flap and split-thickness skin graft. As he was thin built, the hypogastric flap was also thin without much fat. In our procedure, a major portion of the testes was covered by split skin graft so that it would minimally affect spermatogenesis. And penoscrotal and perineoscrotal junctions and a part of scrotum were covered by the hypogastric flap to have a good erectile and sexual function.

The results were very satisfying as he did not have any erectile problems. The flap avoided a contracture occurring at the penoscrotal and perineoscrotal junctions. The split-thickness skin grafts on the testes would probably improve spermatogenesis comparatively. The scrotum was mobile and cosmetically appealing. The couple was psychologically satisfied with their sex life and his wife conceived thereafter. Our results were thus satisfactory from an aesthetic as well as functional point of view.

## CONCLUSION

In our case, the contour of the scrotum is well maintained and sexually the couple is fully satisfied because of the good erection. So we feel that in penoscrotal skin avulsion, penis should be covered with split-thickness skin graft. The penoscrotal and perineoscrotal junction should be covered with a flap cover and the remaining area of the scrotum, the testes and the donor site should be covered with split skin graft. While a variety of methods have been described, the combination of hypogastric flap and split skin graft is one of the best methods to not only have an aesthetic result but also maintain the sexual and testicular function. Long-term fertility will need to be evaluated however.
